# The beginning of becoming a human

**DOI:** 10.18632/aging.205824

**Published:** 2024-05-06

**Authors:** Polina A. Loseva, Vadim N. Gladyshev

**Affiliations:** 1 Division of Genetics, Department of Medicine, Brigham and Women’s Hospital, Harvard Medical School, Boston, MA 02115, USA

**Keywords:** human, aging, 14-day rule, life

## Abstract

According to birth certificates, the life of a child begins once their body comes out of the mother’s womb. But when does their organismal life begin? Science holds a palette of answers—depending on how one defines a human life. In 1984, a commission on the regulatory framework for human embryo experimentation opted not to answer this question, instead setting a boundary, 14 days post-fertilization, beyond which any experiments were forbidden. Recently, as the reproductive technologies developed and the demand for experimentation grew stronger, this boundary may be set aside leaving the ultimate decision to local oversight committees. While science has not come closer to setting a zero point for human life, there has been significant progress in our understanding of early mammalian embryogenesis. It has become clear that the 14-day stage does in fact possess features, which make it a foundational time point for a developing human. Importantly, this stage defines the separation of soma from the germline and marks the boundary between rejuvenation and aging. We explore how different levels of life organization emerge during human development and suggest a new meaning for the 14-day stage in organismal life that is grounded in recent mechanistic advances and insights from aging studies.

## Life as defined by movement

Debates on when human life begins are rooted deep in philosophical history. However, until recently they have been limited by the state of technology. No scientist could have a chance to witness the emergence of a new human life hidden within the uterus, as no methods were available to peer inside and observe it directly.

Natural scientists made several attempts to draw the line between an animated embryo and an unanimated substance. Aristotle, for example, believed that the male embryo acquires a soul on the 40th day of development, while the ensoulment of a female might not occur until the 90th day [1]. However, the only proof of a successful pregnancy that remained generally accepted till the 19th century was the first movement of the fetus, i.e. the quickening. In 18th century England, only movement of the fetus could serve as a reason for pardoning a pregnant woman sentenced to hanging [1].

The wellbeing of an embryo at earlier stages wasn’t much of a concern — neither for the mothers, nor for the scientists. Some researchers would easily sacrifice an embryo or a fetus to turn it into a whole-body preparation or a set of histological sections. A striking example of this attitude was the history of the Carnegie embryo collection. During the first half of the twentieth century, the Carnegie Institution managed to gather several thousand human embryos. Hunting the embryos, researchers would closely monitor women to whom hysterectomy was prescribed. Any removed uterus could procure them with a new specimen — sometimes accidentally (since there were no accurate pregnancy tests yet) and sometimes purposefully, if the woman was asked to become pregnant shortly before the operation [2, 3]. This method hardly caused any public debate, and on its basis the Carnegie researchers worked out a table of human development stages, which has been widely used ever since.

However, quickening is not the best criterion for life. The timing of the first movement varies widely and mostly depends on the mother, not on the fetus [4]. Some women (mostly primigravid ones) have to wait almost till the third trimester of pregnancy to notice the first movements of the baby, whereas others feel it already by the end of the first. A better criterion was needed, but no one urged to look for it—perhaps, because the doctors at the time were unable to modify the process of human development; and the only manipulation they could perform with the emerging life was to take it away.

This manipulation, however, had been widely banned by the end of the 19th century as the Catholic Church declared that an embryo should be considered alive at any stage of development whether “animated” or not [1]. Still, there are several mechanistic obstacles which make this criterion hard to use, especially in clinical settings.

## Life as defined by fusion

The model where every embryo is considered alive implies that the emergence of life equals the emergence of conceptus, i.e. a new cell identical neither to maternal nor to paternal cells. The most evident property gained by this new cell is apparently a unique set of genes. However, a zygote neither has a proper nucleus nor an assembled set of genes. The only RNAs that “work” in the cell supporting protein synthesis after fertilization are those inherited from the egg. The maternal and paternal chromosomes remain tightly packed and align in the center of the cell forming the mitotic spindle for the first division. During the cleavage, chromosomes mix up between daughter cells and so only by the first telophase the new chromosome sets are assembled [5].

One could also speculate that the assembly of the nucleus is not a functional boundary and that the zero point of life should be set when the newly formed genome starts expressing its own genes. Then it should be set even later as the first blastomeres rely on the maternal RNA until days 2-4 when the zygote genome is fully activated [6, 7], although recent studies suggest that the embryonic gene expression may initiate at one cell stage already [8]. However, if the gene activity served as the marker, the timing of the zero point could be different depending on which particular genes one considered crucial for embryo development. For example, the first differentiation event relies on *Cdx2* expression, which is specific for the future trophectoderm and occurs later, around day 5 post-fertilization [9].

This genetic ambiguity wasn’t an issue in the 19th century but would certainly become one now, for example, within a context of an IVF facility. Should every “freshly fertilized” zygote be considered a human being or should embryologists wait for a switch in gene expression?

This is not the only obstacle to consider life beginning at the time of fertilization. Until a certain point in development (which occurs around two weeks, see below), a mammalian embryo can be split into several separate beings. This process occurs naturally, i.e. this is how monozygotic twins are formed. If life begins at the time of fertilization, is it then split into two lives?

**Figure 1:**
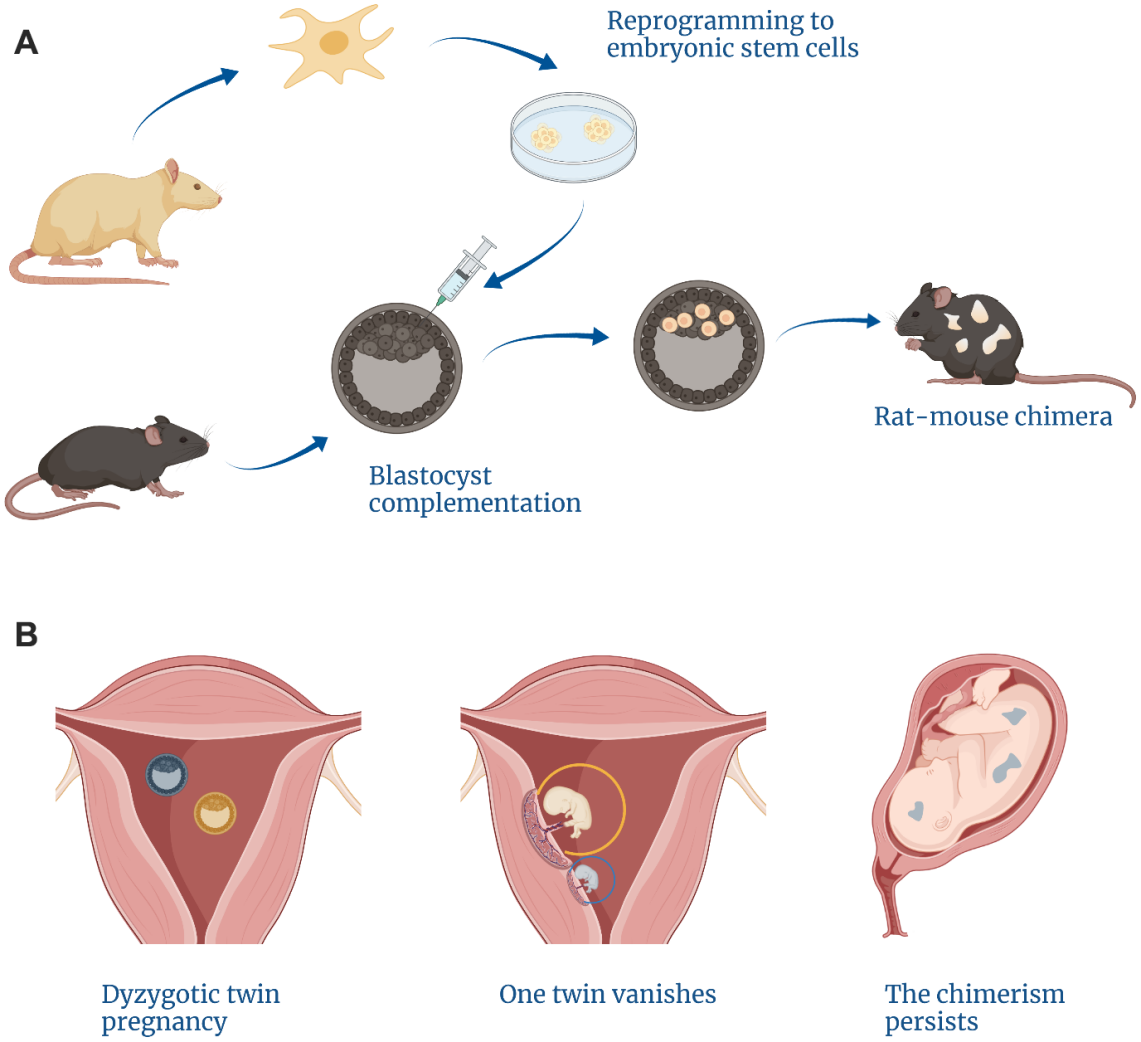
**Examples of cases arising from a lack of self/non-self discrimination within the embryo.** (**A**) Blastocyst complementation methods can give rise to interspecies chimeras. (**B**) The vanishing twin phenomenon is responsible for natural intraspecies chimerism.

The early embryos can also be combined into a single chimeric organism. This process underlies the phenomenon of a “vanishing twin” when one twin embryo merges with the other [10]. Then, the cells of the vanished twin dissipate within the tissues of the persisting twin and may manifest later as simultaneous presence of different blood types [11], Y-chromosome bearing cells within a female organism [12], or the germline within the other twin’s organism (Figure 1B) [13].

Moreover, chimeras can be created from early embryos belonging to different species. This can be illustrated by the reports of various non-primate mammalian chimeras such as rat-mouse chimeras that develop into viable animals (Figure 1A) [14]. Human embryonic stem cells also preserve the ability to survive within non-primate embryos (such as pig [15] or mouse [16]).

One could argue that these observations prove that during early stages of development the embryo cannot discriminate between self and non-self. Although chimeras between closely related species are formed easier than between distant ones (the first human-monkey chimera comprises up to 7 percent of human cells which is much higher than most human-mouse chimeras [16]) and the level of apoptosis in these mixed embryos remains high, the fact that they can result in a viable fetus suggests that the rejection of foreign cells is not too high [17]. This is not surprising since early embryos do not possess a proper immune system and the major histocompatibility complex supposedly does not come into play until later stages [18]. Still, this readiness to accept cells of a different being and even of a different species seems to contradict the idea of life starting at the onset of development.

## Life as defined by self-sufficiency

Secular authorities gradually relaxed the rules. They admitted that an abortion might be justified by social or medical issues before the fetus gains certain physiological self-sufficiency. However, a point where a self-sufficient life begins — and beyond which it should not be acceptable to end it — was not easy to define.

The criteria of death as the endpoint of life are well established though constantly evolving [19]. We are used to define it as a cessation of breathing and heart beating or disappearance of brain electrical activity in a hospital setting [20]. But the same rules do not work the other way around, as the vital organs do not appear simultaneously and develop gradually. For example, the first fetal diaphragm movements (which are not yet a true breathing, however) can be detected as early as 10 weeks of development [21], but the first contractions of what one day will become a heart appear already at the third week (although neither the layers of the heart nor the chambers are formed yet) [22].

In some legislations, medical issues (when there is a significant risk to the mother) justify an abortion up to the very birth of the child. And in most Western countries today women are allowed to ask for an abortion independently of their health risk until a certain point which is officially set at the moment when the fetus becomes viable, e.g. can exist outside the uterus (albeit with the help of doctors) [23]. But this time point is constantly shifting [24].

In the 1970s, at the time of Roe vs. Wade case, it was the beginning of the third trimester (27 weeks), then it became possible to support a baby’s life already at the 22nd week post conception [25]. There have been reports of prematurely born babies surviving even at the 20th week [26]. It may be expected that as neonatology develops further, this threshold will also move—and the zero point of human life defined this way may then depend on the country, the clinic or even skills of a particular doctor. Clearly, all these regulations would need yet another revision once an artificial uterus-mimicking system is established.

Thus, for a long time the law had been protecting only those embryos that could survive independently of the mother and had focused only on the cessation of an established life implanted inside the uterus. All other stages of human development remained inaccessible to doctors. That is, until 1978 when Louise Brown was born [27].

## Fruitful technologies

**Figure 2:**
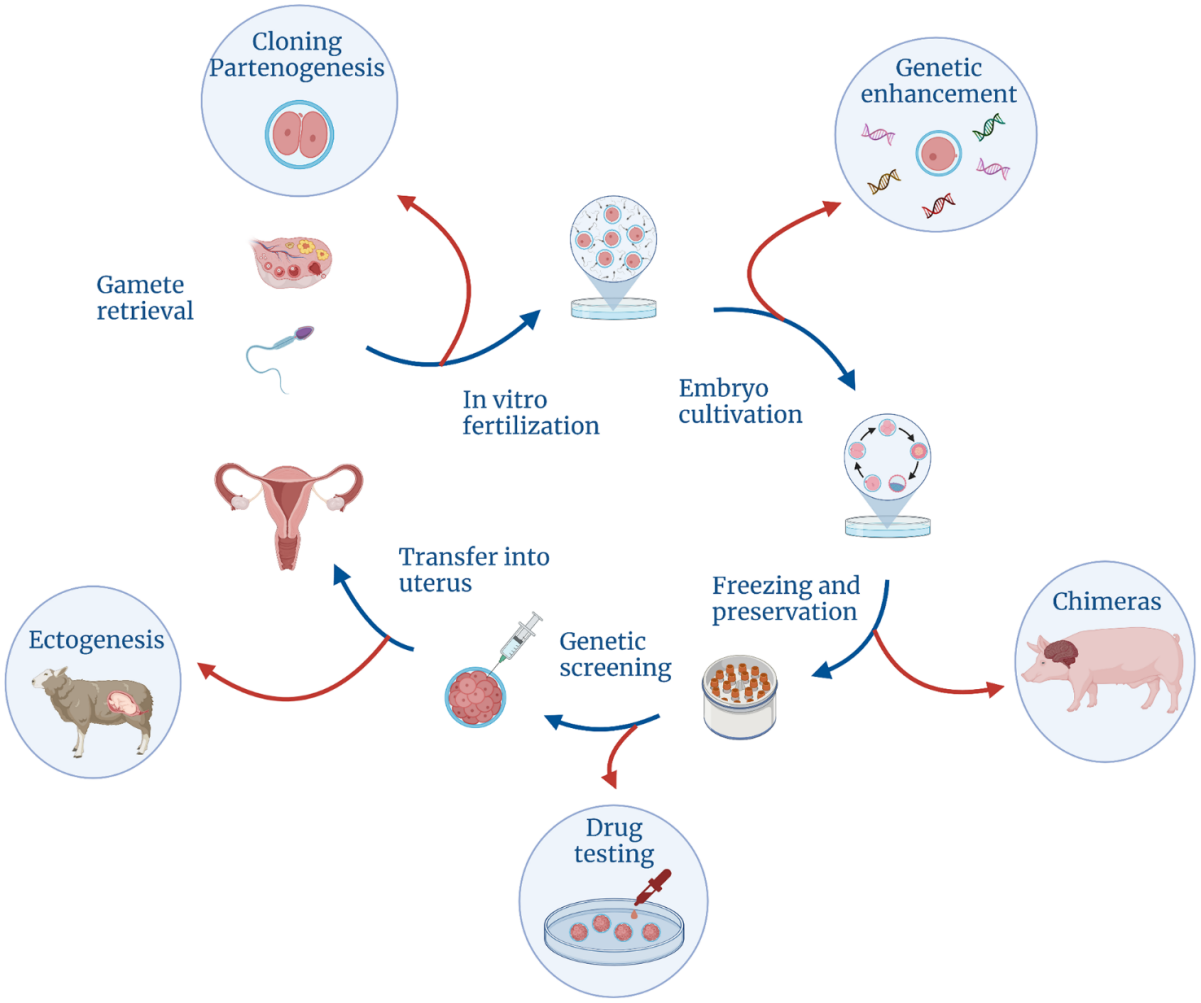
Controversial technologies that could be made possible with the development of different stages of the regular IVF cycle.

Louise’s parents, Lesley and John Brown, were neither seriously ill nor genetically incompatible. However, they could not conceive a child naturally for nine years as Lesley’s fallopian tubes were severely deformed [27]. Multiple operations did not help to clear the blockage, so the attending physician suggested a new option [28]. He called it “reimplantation” of the embryo into the uterus—now we call it “*in vitro* fertilization”, or IVF: here, the sperm and the egg meet inside a Petri dish, the embryo is grown and observed for several days and then transferred to the woman’s uterus (Figure 2).

Louise Brown was born in term and her birth was no different from any other British girl, except for the police guards outside the hospital [29]. The government feared that this case could attract too much media attention—and so it did. After the birth of the first so-called “test-tube baby” (although there was no test-tube actually involved in the process) it became clear that there would be many more as the demand was extremely high. About one in ten UK families at the time was believed to be childless [30].

The Brown couple’s second daughter, Nathalie, who was born four years later, is said to be the fortieth IVF child in the world. As more infertile couples learned that they had a chance to give birth, “*in vitro*” babies appeared in the USA, Australia and India. In 2018, doctors estimated the number of children born after IVF at eight million [31].

Breakthrough technologies often do not arrive alone. The IVF procedure comprises many steps of which fertilization itself is not the hardest one. In order to transfer the several day-old embryo to Lesley Brown’s uterus, the doctors had to learn how to extract egg and sperm cells from the parents, how to keep them viable, how to make them find each other in a dish and then—probably the most important step—how to culture the embryo and check whether it develops normally.

With the birth of Louise Brown, a new era began, in which a doctor could influence not only the life of a person who was already born, but even the development of a person who has not yet been conceived. Embryologists were yet to learn how to freeze and unfreeze embryos, remove single blastomeres or replace particular genes with new ones. But they already foresaw that, sooner or later, it would happen and they realized that opportunities had opened up for even more complex manipulations with human embryos. The fantasy of scientists had started running wild [32]. In the early 1980s, they could already imagine drug testing on embryos, raising children outside the uterus (ectogenesis) or in the body of other animals, parthenogenesis (creating embryo from an egg without involving any sperm), human cloning and—of course!—genetic improvement of people.

One could not envision what the life of a child born after this kind of manipulations could look like and what long-term consequences it could have for his or her wellbeing. How to predict what might happen in the head of a person endowed with only maternal genes? Or a child grown inside a pig womb?

However, childlessness was considered an important issue. There had already been a significant public demand for reproductive technologies. And obviously the development of reproductive technologies would imply refining and testing of basic procedures, which is impossible without experiments on early human embryos. Thus, a compromise was needed between the desire to give a chance to exist to those who were deprived of it, and the risk of ruining this existence.

New technologies came along loaded with new responsibilities. The scientific community had to think seriously about how to handle the emerging new opportunities. Therefore, in Great Britain, in 1982, the Committee for the Study of Human Fertilization and Embryology was gathered. It is also known as the Warnock Committee, after its chairman, writer and philosopher Mary Warnock. The committee was tasked to draw a line between acceptable and unacceptable embryonic experiments. For two years, members of the Committee had been interviewing about 300 doctors and embryologists and studying the opinions of almost 700 fellow citizens. Their verdict came out in the year of 1984—when both “test-tube” Brown sisters were well up on their feet [32].

## Life as defined by the Warnock Committee

By 1984, no experiments were possible without informed consent of the person being experimented on. However, an embryo cannot be informed and has no means to give consent. Should this serve as a basis for all embryonic experiments to be banned, depriving thousands of infertile couples of a chance to reproduce and leaving many developmental pathologies understudied and incurable? Or, vice versa, should an embryo be considered other than a living person, given that it is not conscious and does not possess most of human properties?

For the first time, a strict answer was required to the question of when a person’s life begins. However, this was exactly the only question that the Warnock Committee did not answer. It was clear from the very beginning that no answer would equally suit everyone. That’s why the Committee proceeded with its verdict stating that the question of the beginning of human life does not have only one answer [32]. Although it appears to be a question “of fact susceptible of straightforward answers”, it stated, we hold that the answers to such questions in fact are complex amalgams of factual and moral judgements”, it claimed. This kind of formulation would not help to direct the research. Instead, the Committee brought in another question, an instrumental one: what is the developmental stage at which it is justifiable to destroy a human embryo if something goes wrong with it?

The Committee had a predecessor, the US Department of Health, Education and Welfare Ethics Advisory Board, that had attempted to solve a similar problem several years before. In its landmark report of 1979 termed “HEW Support of Research Involving Human *In Vitro* Fertilization and Embryo Transfer” the Board suggested that no embryo should be grown in culture past the 14-day stage [33]. This choice was said to be motivated by the timing of the implantation. In fact, the board members produced polar views on the topic and the 14th day was chosen as an arbitrary point when the embryo should have been already implanted in the uterus but could not have developed any differentiated tissues.

In contrast to the report of 1979 that was never put to clinical practice, the Warnock Committee’s decision proved to be very influential. The Committee chose the same time point for the boundary but produced additional arguments to that point. It decided to treat the issue of experimenting with embryos in the same way as with any other people. The ethics of clinical trials suggests that the benefit should outweigh the suffering. But since we cannot measure the intensity of an embryo’s suffering, the only period when we can be sure that the benefits are higher is when there is no suffering at all.

However, it was unknown when exactly the embryo acquires the ability to suffer. So, the Committee based its decision on the point when the first signs of the nervous system appear in the embryo, which is the 17th day of development. Still, the Committee admitted that it could not be the finest estimate, and as our knowledge on human development is refined, this border could be shifted further. So, not willing to take this risk, the Committee decided to set the boundary a few days earlier. The result was 14 days. Later, Mary Warnock confessed that a cutoff of 14 days was not the only option [3]. It could have just as well been 13 days or 15, nothing would have changed. She chose 14—”simply because everyone can count up to 14; a fortnight is a good, memorable number, and records can be kept week by week”.

Now that we know much more about the development of the human brain, it is obvious how arbitrary that cut-off was. A recent report states that no neural progenitors can be found in 16-19 day human embryos [34]. It is also well known that synchronized impulses of neurons in the peripheral nervous system can be detected not earlier than the second month of development. And still, this does not mean that the embryo is already capable of feeling the pain. Research suggests that the nervous system of an embryo is fully “ripen for suffering” only by the 19th week after fertilization [3]. What can be mistaken for the nervous system in a 14-day-old embryo is just a marking, a blurry shadow of what one day will become a full-grown brain and a spinal cord. So if anyone were to use this criterion for defining a boundary for human life, they would also struggle from multiple inconsistencies.

However, the Committee’s report stated clearly enough that no criterion could be universal and comprehensive. It was a compromise that should have been made so that everyone felt listened to—those who upheld religious principles, those who came up with new reproductive technologies, and those who were waiting for their chance to give birth to a child.

Yet, the 14th day stage still attracted the attention of biologists, as it is accompanied by more obvious transitions, which mark a fundamental developmental stage.

## Life as defined by uniqueness

**Figure 3:**
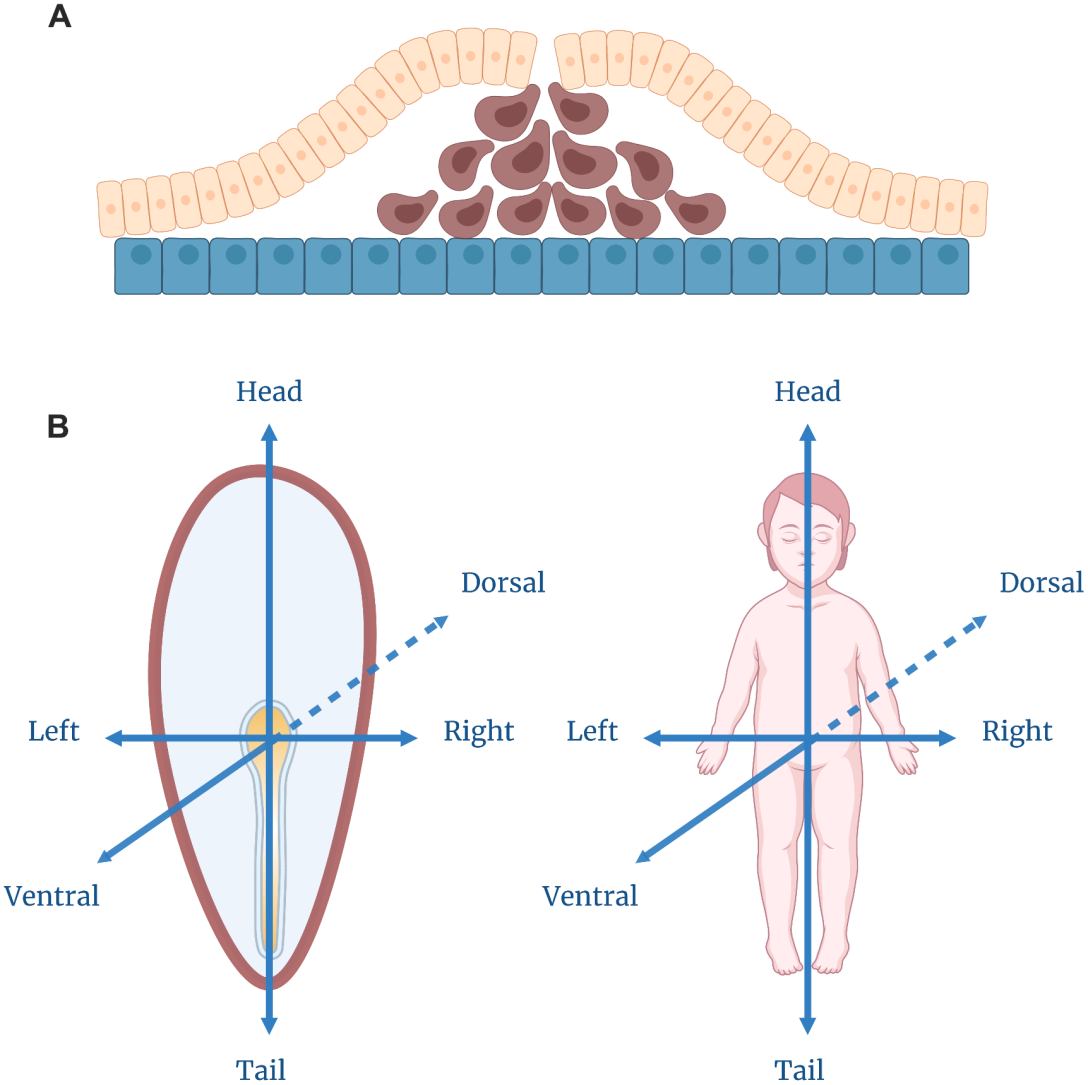
**Gastrulation.** (**A**) Cell movement during the gastrulation process. The yellow layer depicts the epiblast, and the blue layer — the hypoblast. The cells in brown are migrating to form, first, the endoderm, and later, the mesoderm layers. (**B**) Axis specification within a gastrulating embryo which corresponds to the ultimate body plan of a human being.

The first two weeks of human development yield a single-layer cell disc surrounded by several bubbles of germ membranes. By the third week, a process begins that makes this disc more human-like (Figure 3). This process is gastrulation—”truly the most important time in your life”, according to a quotation attributed to the embryologist Lewis Wolpert [35].

There is one important property that distinguishes an archetypal human from a cell disc: a human being consists of three layers. One can imagine the entire human body as a three-layer sandwich wrapped around the hollow tube (the intestine). The gastrulation does not provide the embryo with a proper intestine, but it results in a three-layer disc homologous to the gastrula stage of vertebrate development.

The resulting structure does not resemble a human body as we are used to seeing it. However, an experienced biologist can tell for sure what it will look like as it grows up (Figure 3). Where the first thickening appeared, the posterior end of the body will subsequently develop, the opposite side of the disk will form the anterior head structures [36]. The migrating cells point to the ventral side of the body, while the remaining outer cells will turn into the dorsal side. In the center of this dorsal region, cells will become the nervous system: they will continue to divide and sink under the upper layer, where they form the brain and the spinal cord. In a week, we will get a prototype of a typical vertebrate animal with the head opposed to the tail and the neural tube, the notochord and the intestine aligned along the dorsal-ventral axis—a structural plan that is the same for humans, lizards, frogs and fish.

However, the more patterned the embryo gets, the harder it is to split it into several parts. During the previous stages of development, while the embryo was nothing but a clot of uniform cells, it could be easily divided into two (or more) separate clots which would continue to develop independently and could grow into identical twins—each with the complete set of their own organs. Then, each morphogenetic event makes this kind of division riskier. After the anterior-posterior axis is established, it is no longer possible to split the three-layer disc into equal parts so that each harbors a full set of organs (at least there has not been any reports of that kind [37]). The only option for those embryos who are “late” to branch out a sibling and split beyond the 14th day is to share one body and become Siamese twins. The sanctions for being late are harsh: often one or both twins are not viable [38].

Thus, the 14th day of development turns out to be a paradoxically significant event. At this point, on the one hand, the future person gains the shape and the features shared by all vertebrates. On the other hand, this is where its individual development begins—there will never be another one with the same genes. Looking for a safe time point when the embryo is incapable of feeling pain, the Warnock Committee settled upon a date when the future human being actually gains its uniqueness. This is probably one of the main reasons why the “14-day rule” was widely accepted and strictly followed for the following 37 years.

## Pushing the frontiers

The report of the Warnock Committee suggested that “no live human embryo derived from *in vitro* fertilization, whether frozen or unfrozen, may be kept alive, if not transferred to a woman, beyond fourteen days after fertilization, nor may it be used as a research subject beyond fourteen days after fertilization” [32]. By the 15th day, the embryos should be destroyed. And this recommendation (the so-called “14-day rule”) was soon accepted—not only by the British government, but also by other countries [39]. Somewhere, like in Japan or Belgium, it was included in the law, in some other countries (like in the United States) human embryo research is entirely deprived of federal funding. Later, the rule became a part of the International Society for Stem Cell Research recommendations: in the updated version it prohibited to culture human embryos beyond the primitive streak stage irrespective of how many days it had actually spent in a dish [40].

In 1984, this rule did not suit everyone. The Warnock Commission was accused of being too utilitarian and referring to the technical aspects of child creation while ignoring the moral ones [41]. Still the embryologists seemed to fit well in the new rules: the handicap of two weeks was more than enough for their current research purposes. At that time, none of them would have been able to cross this line—the embryos still could not survive on their own for so long.

The main issue, however, was not just the food and oxygen—those could be easily provided *in vitro*. A mammalian embryo depends very much on the mother’s organism as a source of spatial signals which coordinate the topology of its development. Endometrial cells provide the embryo with mechanical support and also produce signaling factors that influence the specialization of extraembryonic tissues which in turn are involved in the gastrulation process [42, 43].

Therefore, at the time when Mary Warnock proposed her “memorable number”, it meant no offense to any ongoing research. So, it was not an actual compromise, it was a limitation that no one had a chance to overcome—though it was expected that it could be possible in the future. The time for a real compromise was yet to come.

Since then, technologies have changed beyond recognition. Embryologists gained access to synthetic polymer substrates, three-dimensional scaffolds, 3D printers, microfluidic devices and state-of-the-art incubator-maintenance systems [44–46]. Many more options are available now, making conditions within a culture much more similar to the maternal womb. Hence, the more support the embryo gets from an artificial “mother”, the further it can develop.

The first challenge to the 14-day rule arose in 2014 when a group of scientists used a morphogen to induce a cluster of human pluripotent stem cells to form several embryonic cell types [47]. The resulting structure did not resemble the embryo morphologically; however, it recapitulated the three-layer pattern of organization—and that’s why this kind of structure was termed a gastruloid. Later, in 2016, it became possible to cultivate a real human embryo for a longer time, imitating post-implantation stages [48, 49], and in 2018 a synthetic embryo (a blastoid) was assembled from stem cells [50].

Of course, none of those embryo-like creatures were capable of self-sufficient development. Though the gastruloids demonstrated a clear distinction (verified by gene expression) between the outer, inner and middle layers, they lacked extraembryonic tissues and did not recapitulate the structural plan of the early human embryo. Only in 2019, microfluidic technologies offered an opportunity to induce anterior and posterior structure formation within an embryo [45]. However, the whole anterior-posterior patterning was still unachievable. While the embryo-like structures showed a clear dorso-ventral polarity, each of them developed either anterior- or posterior-like features.

In 2021, it became possible to mimic the whole early human development *in vitro*. Three independent groups managed to create blastoids—blastocyst-resembling structures—from a homogenous pluripotent stem cell culture [51–53]. Blastoid generation provided the artificial embryos with extraembryonic cells, which could be useful to recreate implantation and post-implantation developmental stages *in vitro*.

Another breakthrough achievement of 2021 got us closer to an artificial womb. A group of scientists perfected the long known technology of *ex utero* whole embryo culture [54] and succeeded at growing mouse embryos in a bioreactor up to the 11th day of development [46]. At this stage, embryos develop beating hearts, functional circulatory systems and partially developed limbs. The key to this advance was the development of a unique ventilation and atmospheric pressure maintenance system, which supplies stage-adjusted oxygen levels, uses placenta-derived serum and protects embryos from deforming. To further extend the period of *in vitro* embryogenesis, it will be important to develop either a blood supply system or an artificial placenta. By analogy to mouse embryos, it should be possible to grow human embryos well beyond the 14-day threshold.

What sounded like alarming fantasies in the 1980s, became scientific reality in 2021. So, the time has come for a true compromise between ethical issues concerning human embryo experimentation and scientists peering deeper inside human development and reproductive technologies.

## Post-14 days

So far researchers have faithfully destroyed the results of all these experiments—at least, every experimental report on the topic emphasized the time point beyond which all the cultivation procedures were stopped. However, the very fact that long-term embryo culture became possible triggered a new round of discussion in the scientific community. Since 2016, at least one article appeared every year proposing to move the boundary for experiments considered acceptable [55]. Initially scientists simply reminded that the 14-day rule was not meant to act like a moral dogma. They also acknowledged that since 1984 we have learned a lot about the development of the human nervous system and now one can be sure that after the 14th day the embryo will remain “insensitive” to experiments for a long time [56]. More recently, they began to discuss the practical need for such experiments [57].

Born at the end of 2018, the first genetically modified children, like the Brown sisters, launched another revolution in medicine. The scientific community condemned Jiankui He, who carried out this procedure, however, it became obvious that the range of reproductive technologies had significantly expanded [58]. Modern techniques make it possible not only to conceive a child *in vitro* but also to manipulate its genes—before they start forming a new person. In order to find out whether newly introduced genetic changes affect human development, we will need to observe the embryo for a longer time. Two weeks might not be enough, given that at that stage the embryo does not even harbor proper tissues, let alone organs or body parts.

Each subsequent publication brought more arguments and more proposals concerning new possible boundaries. Some suggested the moment when the nervous system begins to be developed—a time point which somehow aligns with the first contractions of the heart (also a symbolic sign of life), making it 22 days post fertilization [3]. Others urged to push the boundary further, to the point when the first sensitive neuron progenitors appear which will later be able to transmit a pain signal [59]. This moment corresponds to days 29-31 of development. Some scientists stand by the mark of 28 days arguing that the later stages of development can be studied to a sufficient degree on abortive material [56]. Before day 28, however, abortions are usually not done, therefore, our knowledge of what happens between days 14 and 28 days is very limited.

Finally, there are proponents of individual approaches—those who propose making specific decisions after considering the direct pros and cons of long-term embryo cultivation within each particular experiment [3]. This is similar to what happens now with genome editing—in this area, experts are also inclined to make individual decisions depending on how safe and justified they find this or that method of “gene fixing” [60].

Mary Warnock lived to see new disputes arising around her brainchild. She repeatedly spoke out against moving the border she had suggested back in 1984 [61]. The main issue, according to her, was not just the potential trauma that scientists could inflict on embryos, but the attitude of society to reconsidering the compromise. She feared the strengthening of pro-life movements “who do not seem to realize how intensely pro-life IVF itself has always been”. Warnock suspected that attempts to revise the 1984 decision would allow opponents of IVF to claim that the “14-day rule” was not a true compromise and was suggested simply because scientists did not have the technical ability to break it. In some ways, of course, this claim might be true.

In 2021, ISSCR released an update to its recommendations concerning stem cell and embryo experiments [62]. After having revised the regulations and considered the new scientific evidence, the Society canceled the 14-day rule. There is no longer a clear boundary between an allowable and an unacceptable duration of embryonic life *in vitro*. Now the experiments, including long-term embryo culture, fall into the “category B” of regulations, which implies that a special committee consisting of embryologists, bioethicists and lawyers has to review a proposal for each experiment separately and work out an individual decision for each case.

It is yet to be seen whether these committees will dare to overcome the 14-day rule or will stick to the traditional point of view. Unfortunately, Mary Warnock will not be able to see how this story unfolds as she passed away in 2019. However, as the debates around the 14-day rule grow harder, other fields of biology bring unexpected insights on what happens inside an embryo at this very point of development.

## Life as defined by aging

Another way of delineating life is not by the way it begins but by the point when it ends. One can perceive life as a one-way journey ending with death. From this point of view, an organism can be considered alive if it is constantly moving towards death. In a way, living can be considered synonymous to aging.

Aging lacks a proper definition itself [63]. It is often described as age-related accumulation of deleterious changes within an organism, functional decline, continuation of development, or other processes. But these ways of describing aging do not necessarily translate to reliable biomarkers [64]. So most often different proxies are used, such as particular features of damage accumulation, omics-based clocks that measure biological age (e.g. epigenetic aging clocks), overall disease burden or growth of mortality risk [65, 66].

Until the very end of the 20th century, this life-aging parallel was of no use to embryologists since it was impossible to spot any signs of aging within an embryo—even after technologies were developed to culture it *in vitro*. Only recently, after various robust molecular biomarkers of aging have been established, we can look closer at the early stages of development to find out when the first hallmarks of aging appear.

First, we know for sure that certain features of aging can already be seen after several weeks of development. A prominent example of those are single nucleotide variations. More than a third of point mutations (including oncogenic ones) that arise during the whole life of a neuron are acquired prior to birth starting possibly before neuron progenitors are specified [67]. Thus, if one considers accumulation of mutations as a feature of aging, life should begin somewhere at the first divisions of a zygote—with the DNA polymerase making its first mistakes.

Another common feature of aging is the growing risk of mortality. It has been known for a long time that this risk is minimal around the onset of reproduction. This would agree with the idea of aging beginning at the completion of development. However, more recently it was shown that the minimal mortality is around the age of 9, i.e. before humans can reproduce [68]. Moreover, studies revealed that even at this point and earlier, the “true” aging-related mortality is masked by the high early life mortality associated, instead of aging, with developmental errors and purifying selection for deleterious mutations [68]. The time when the aging-related mortality begins to show up was really hard to determine, although it was thought that it emerged somewhere in the first months of development.

At the same time, one could argue that aging cannot start right after fertilization as there should exist some mechanism to reset the age of the gametes [69]. Those have been shown to accumulate various aging-related features (shortened telomeres, aggregated proteins, altered cytosine methylation in the DNA, etc.) [70]. Hence, it should take some time to get rid of those hallmarks of aging, and this process could possibly occur after fertilization. Indeed, it was shown that telomeres do actually elongate in the first days of embryonic development [71]. It was also shown that in certain animal species a process of active proteolysis takes place after fertilization sparing the blastomeres from potentially toxic aggregates [72, 73].

Recently, direct assays to estimate the biological age of early embryos have been devised. This age can be measured by using epigenetic aging clocks, which are biomarkers that reflect changes in DNA methylation at certain sites within the genome [74]. Application of epigenetic aging clocks showed that the age of blastocysts, embryonic stem cells and induced pluripotent stem cells is close to zero [75].

However, it turned out that the lowest age, which was termed the ground zero point, does not coincide with fertilization or activation of the embryo’s own genome [76]. In mice, this state from which it is thought aging begins corresponds to approximately 7-10 days after fertilization. In humans, this state is predicted to be achieved at a similar stage—after the implantation, around gastrulation [77].

Interestingly, this ground zero point of human aging falls closely to the phylotypic stage of vertebrate embryogenesis. According to the hourglass model of evolution, the earliest developmental stages are not the most conservative ones [78]. The period when all vertebrate embryos look the most similar to each other (and express genes that are evolutionarily the oldest [79]) corresponds to the gastrulation and neurulation stages after which the diversity grows both in morphology and gene expression.

**Figure 4:**
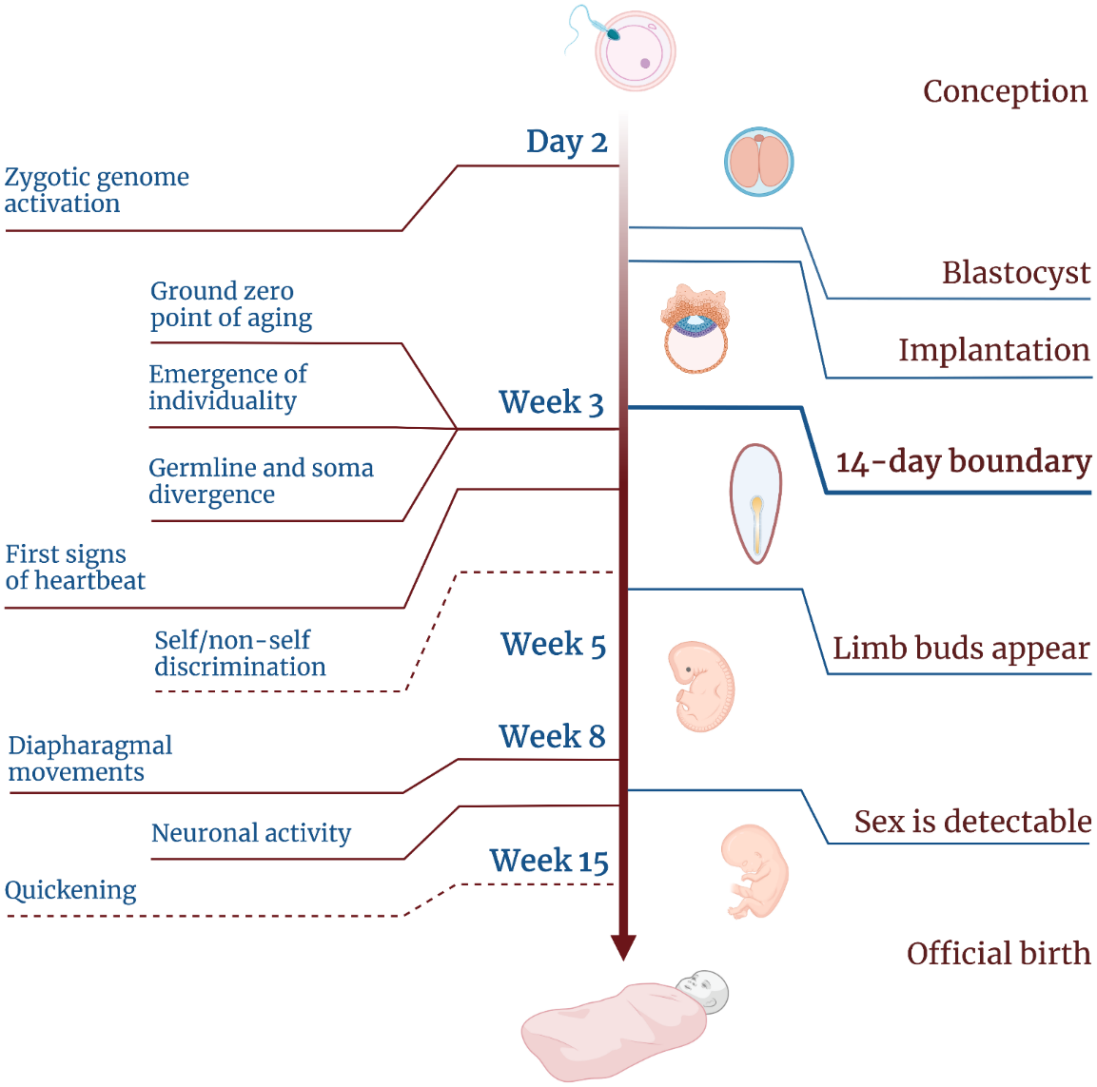
**Timeline of human embryogenesis showing the developmental stages and the emergence of different features of life.** Dashed lines indicate two features for which the timing is uncertain.

Thus, it is likely that the ground zero point of aging is grounded in evolution and conserved across vertebrates. In humans, it would fall on the third week after an egg meets a sperm—at or right after the boundary set by Mary Warnock and her colleagues in 1984 (Figure 4).

There is one more event that takes place at the same time—it is the specialization of human primordial germ cells [80]. These cells share the ground zero point of aging with all the rest of the embryo; however, later on they age differently, as germ cells can age only as much as to be able to completely rejuvenate in the next generation, whereas somatic cells can age much more if this maximizes fitness. Thus, this embryonic stage can be viewed as the beginning of somatic life, or the birth of soma—the part that constitutes most of the body and is the subject to development, aging and death. This corroborates the view of the 14-day stage as the ground zero point of aging. So, if aging may be considered an indispensable feature of life—at least for somatic cells—then this stage can be viewed as the starting point of life for the soma.

## 14++

The lack of scientific consensus about the beginning of life brings us to a paradoxical situation. The last ISSCR decision implies that—at least until the next update of its recommendations—whichever point we consider the beginning of human life and the point when we accept the responsibility for this life will not coincide. The former is completely left out of scientific discussion (as there is no correct way to define it), and the latter will be set each time individually.

We are, once again, in a situation where the scientific and the ethical considerations diverge. The challenge to set the new boundary for embryo experimentation will require a broad discussion, not only among embryologists, but also including bioethicists, lawyers and representatives of various social groups. This new boundary might not only differ between countries but also evolve along with the attitude of society towards such experimentation that will surely change over time (which can be exemplified by the recent overturning of the Roe v. Wade case).

The science of human development, however, takes its own path. Wherever the boundary would be set next, the scientists are left with biological facts gathered around the previous boundary—as well as the need to further conceptualize it. Forestalling future experimental and conceptual advances, the Warnock Committee was wise enough to point out a unique stage of human development. Whether this stage remains a legal and widely accepted boundary for experimentation or not, it now becomes a grand scientific question in itself, as it emerged as a turning point in embryogenesis.

From what we know at the moment, this stage holds several important features (Figure 4). First, it marks the endpoint of rejuvenation, which supposedly starts some time after fertilization [75]. Second, it marks the ground zero moment, the beginning of the aging process at the molecular level. Third, this stage sets the boundary for uniqueness. Prior to this stage, individual embryos may be easily combined and split without impact on further development.

However, it is difficult to define this stage in terms of human life as there are no good criteria for life itself. All the definitions so far have proven to be inconsistent or controversial when it comes to practice and clinical use. So instead of defining human life as a whole we may focus on stages that mark the emergence of different levels of life organization. Thus, one could consider a cellular level (when an embryo can be seen as a living system), an organismal level (a point when a group of cells can be seen as a foundation for a new organism) and a human life level (a boundary which marks the recognition of an embryo as a human being).

These levels of organization can emerge at different time points or some of them may be found to coincide. The basal cellular life level may have no boundary at all (until we learn to build synthetic cells) as there is no point when the gametes or the emerging embryo stop being a living system at least in terms of metabolism. The boundary for the upper one, a human being level, will be a subject to further debates involving scientists and bioethicists.

What we are most interested in is the intermediate one, the organismal level. The timing of the transition from a bunch of cells to an organized structure is not evident, although we suggest that the 14-day stage could be a good candidate for this point. This is the stage where the embryo begins to show signs of self/non-self discrimination. The cells are organized in layers that form a draft for the body plan, and this structure cannot be easily split into parts. Finally, all these cells are done with the rejuvenation processes and some of them—namely the soma—have already started to age. So this layered structure can now be seen as a living organism which does not have all the human attributes yet (and may not even become a human, for example, in case it bears any lethal mutations or chromosomal abnormalities), but it has already acquired its proper boundaries and has started a new aging-rejuvenation cycle.

However, recent studies on synthetic embryos add a new dimension to this hypothesis. These advances open up new opportunities to witness the emergence of organismal life *in vitro*, to explore the molecular processes underlying this transition and to find out whether it is indispensable for human development.

## Becoming a human *in vitro*

Until recently, the 14 day stage was impossible to recapitulate *in vitro*, even in animal models. During the breakthrough experiment involving mouse ectogenesis the embryos started their development inside a womb and were removed to continue their growth *in vitro* only after implantation. However, several groups have now managed to overcome this limitation: their studies revealed that mouse embryo development can be recapitulated up to day 8 which is beyond gastrulation [81, 82]. And during the last year similar advances have been made in the case of human embryos [83–87].

First human embryo models, e.g. gastruloids, neither fully recapitulated the features of a real embryo nor could really be called human embryos. Later on, several studies were reported that aimed to create synthetic embryos out of pluripotent cells. This resulted in the generation of blastoids that shared a certain degree of similarity with natural blastocysts, however, it was not clear whether they were capable of transitioning to further developmental stages.

In 2023, several reports were published describing complex models which, according to claims of their creators, harbored certain types of cells and structures characteristic of the 14-day stage. For example, Ai et al. reported the presence of primitive streak-like progenitor cells in their E-assembloids [87], Pedroza et al. detected primitive streak cells within their extra-embryoids [86], while Oldak et al. described the emergence of germ-line progenitor cells, amnion, yolk sack, chorionic cavity and even the rudimentary umbilical cord in their stem cell embryonic model (SEM) [83].

None of those experiments lasted longer than 14 days, nor did the researchers aim to grow their synthetic structures past the primitive streak stage. However, given the advances in mouse ectogenesis, one could imagine that next time someone might attempt a longer-term cultivation experiment. Thus, a question arises whether these cellular constructs can be termed embryos, treated like real embryos and be subject to the restrictions imposed by the Warnock Committee or the latest ISSCR guidelines.

Each of those embryonic models had certain differences from real human embryos of the corresponding Carnegie stage. Some of them lacked certain cell types, others had a different shape. Strikingly, all of the models lacked fully developed extraembryonic tissues. Though most of the synthetic embryos managed to develop an amniotic cavity and some of them progressed further to grow a yolk sack, none had a full-scale trophoblast (which may be due to the fact that the experimental setup did not include the proper uterine tissue). Thus, these synthetic embryos are incapable of self-sufficient development, even if transferred to a surrogate uterus, although this does not mean they won’t be able to progress further in laboratory settings. So, new criteria are needed to distinguish between a living human embryo and a synthetic bunch of cells.

After the publication of these reports, a group of Cambridge researchers, including scientists and bioethics experts, set out to elaborate on those criteria. The newly-formed Governance of Stem Cell-Based Embryo Models (G-SCBEM) project aimed to develop a new framework on embryo research that would incorporate the issue of dealing with synthetic embryo models.

While this work is still in progress, Rivron et al. suggested a new definition of an embryo [88]. According to it, to be considered a human embryo a model should constitute “a group of human cells supported by elements fulfilling extraembryonic and uterine functions that, combined, have the potential to form a fetus”. This formula does not require the embryo candidate to share a similar structure with a real embryo, nor to harbor all the characteristic cell types.

Moreover, this definition does not imply that the embryo should have passed all the developmental stages from day 1 to day 14. Independently of their life history, a group of human cells can be considered an embryo as soon as it gains the possibility to perform all the necessary functions. This reflects what the researchers saw in their embryo models: some of the synthetic structures skipped the blastocyst stage and progressed right into the days 9-10 of a normal development.

This brings us to the next question: can the 14-day stage be skipped as well by bringing together several embryonic layers? This could constitute not just a technical challenge, but also a test of a key hypothesis—whether this time point is a crucial boundary for the self-organization of cells within a developing embryo. It may happen that producing a synthetic embryo of later developmental stages will turn out to be an overly sophisticated task, as it will require producing and assembling too many cell types. However, if a post-14-day embryo is finally produced (at least in mice), it would be interesting to probe its viability. In case it proves to be capable of a full-term development, that would mean that the ground zero point of aging can be bypassed.
